# Effect of caspase inhibitors on hemodynamics and inflammatory factors in ARDS model rats

**DOI:** 10.1038/s41598-024-67444-5

**Published:** 2024-07-15

**Authors:** Aiming Liu, Fei Tian, Yaqing Zhou, Zunguo Pu

**Affiliations:** 1 Department of Critical Care Medicine, Affiliated Haian Hospital of Nantong University, No. 17 Zhongba Middle Road, Haian, Nantong, 226600 Jiangsu China; 2 Department of Medical Imaging, Haian Hospital of Traditional Chinese Medicine Affiliated to Nanjing University of Chinese Medicine, Nantong, Jiangsu China

**Keywords:** Apoptosis repressor with a caspase recruitment domain, Acute respiratory distress syndrome, Pulmonary hypertension, Hemodynamics, Inflammatory factors, Biological techniques, Cell biology, Molecular biology, Systems biology, Medical research, Respiratory tract diseases

## Abstract

To study the effects of caspase inhibitors on hemodynamics and inflammatory factors in acute respiratory distress syndrome (ARDS) model rats. Sixty healthy male Wistar rats were randomly divided into three groups, namely, the control group, ARDS group and ARDS + Caspase inhibitor group, with 20 rats in each group. The control group was intraperitoneally injected with 2 mL/kg saline, and the ARDS model group was established by intraperitoneally injecting 4 mg/kg Lipopolysaccharide (LPS), ARDS + Caspase inhibitor group was adminstered 20 mg/kg caspase inhibitor after intraperitoneal LPS injection. Changes in pulmonary arterial pressure (PAP) and mean arterial pressure (MAP) at 6 and 12 h before and after administration were recorded. Moreover, arterial blood gas was evaluated with a blood gas analyzer and changes in the partial pressure of O_2_ (PaO_2_), partial pressure of CO_2_ (PaCO_2_), partial pressure of O_2_/fraction of inspired O_2_ (PaO_2_/FiO_2_) were evaluated. In addition, the lung wet/dry weight (W/D) ratio and inflammatory factor levels in lung tissue were determined. Finally, pathological sections were used to determine the pulmonary artery media thickness (MT), MT percentage (MT%), and the degree of muscle vascularization. The pulmonary arterial pressure of rats was determined at several time points. Compared with the control group, the model group had a significantly increased pulmonary arterial pressure at each time point (*P* < *0*.01), and the mean arterial pressure significantly increased at 6 h (*P* < 0.05). Compared with that of rats in the model group, the pulmonary arterial pressure of rats in drug administration group was significantly reduced at each time point after administration (*P* < 0.01), and the mean arterial pressure was significantly reduced at 6 h (*P* < 0.05). The arterial blood gas analysis showed that compared with those in the control group, PaO_2_, PaCO_2_ and PaO_2_/FiO_2_ in the model group were significantly reduced (*P* < 0.01), and PaO_2_, PaCO_2_ and PaO_2_/FiO_2_ were significantly increased after caspase inhibitor treatment (*P* < 0.05 or 0.01). The levels of the inflammatory mediators tumor necrosis factor-alpha (TNF-α), interleukin-1β (IL-1β) and interleukin-6 (IL-6) in the model group were significantly higher than those in the control group (*P* < 0.01), and they were significantly decreased after caspase inhibitor treatment (*P* < 0.01). In the model group, pulmonary artery MT, MT% and the degree of muscle vascularization were significantly increased (*P* < 0.05 or 0.01), and pulmonary artery MT and the degree of muscle vascularization were significantly reduced after caspase inhibitor treatment (*P* < 0.05 or 0.01). Apoptosis Repressor with a Caspase Recuitment Domain (ARC) can alleviate the occurrence and development of pulmonary hypertension (PH) by affecting hemodynamics and reducing inflammation.

## Introduction

Acute respiratory distress syndrome (ARDS) is a serious lung condition caused by intra- and extrapulmonary factors and is characterized by diffuse damage and increased pulmonary capillary permeability. Pulmonary edema, hyaline membrane formation and pulmonary atelectasis as the main pathological changes and progressive respiratory distress and refractory hypoxemia are the main clinical features^1^. ARDS is a common acute and critical condition^2^. As the disease progresses, multiple factors may alter the fragile balance of pulmonary circulation, making ARDS a recognized cause of pulmonary hypertension (PH) and right ventricular failure^3–5^, with a morbidity and mortality rate as high as 68.5%. The pathogenesis of PH in clinical ARDS patients is a complex, multifactorial process that includes hypoxia, hypocapnia or hypercapnia, vascular compression due to edema or pulmonary fibrosis, increased vasoconstriction and remodeling, thrombosis and pulmonary embolism, elevated alveolar pressure and increased intrathoracic pressure associated with positive end-expiratory pressure (PEEP)^6,7^. During these pulmonary vascular changes, hemodynamic factors may be influenced by differences in overall fluid load.

Apoptosis repressor with a caspase recurrence domain (ARC) is an important endogenous inhibitor of apoptosis discovered in recent years. It is mainly expressed in terminally differentiated tissues, such as the myocardium, skeletal muscle, and brain, and its expression level is significantly increased during hypoxia. In contrast, ARC is not expressed or slightly expressed in nonterminally differentiated tissues^8,9^. At present, research on ARC mainly focuses on heart diseases, and studies have shown that ARC inhibits myocardial cell death and protects the myocardium from the induction of apoptosis^10^. However, in recent years, studies have found that normal pulmonary vascular smooth muscle cells express a small amount of ARC. When ARC-knockout rats are exposed to chronic hypoxia, the degree of pulmonary artery pressure (PAP) increase is significantly reduced, pulmonary vascular smooth muscle cell apoptosis is increased, and vascular wall proliferation and remodeling are reduced^11^. To better understand the interdependence between the pulmonary and vascular systems, this study established an ARDS rat model by LPS administration. The rats were intraperitoneal administered the caspase inhibitor and behavioral changes were observed after model establishment. The changes in the partial pressure of CO_2_ (PaCO_2_), oxygenation index (PaO_2_/FiO_2_), the lung wet/dry weight (W/D) ratio and inflammatory factors in lung tissue before and after modeling were evaluated to provide a theoretical basis for the treatment of PH in ARDS.

## Materials and methods

### Laboratory animals

Male Wistar rats, 8 weeks old, body mass 180–220 g, were provided by Changsha Tianqin Biotechnology Co., Ltd, Animal Production Certificate No.: SCXK (Xiang) 2019–0014. All rats were housed in a 12 h/12 h alternating light and dark room with a room temperature of 25.0 ± 1.0 °C and relative humidity of 50.0 ± 10.0%. The rats were fed standard chow and drinking water. All rats used in this research were raised and used in accordance with the ARRIVE (Animal Research: Reporting of In Vivo Experiments) Guidelines on the Use of Laboratory Animals^12^, were approved by the Institutional Animal Care and Use Committee of Nanjing Medical University at January 10,2022 (IACUC2201020) and were in accordance with the guidelines of laboratory rats research at Nanjing Medical University.

### Experimental reagents and instruments

*LPS derived from E. coli* (L2008) was purchased from Sigma. The rat tumor necrosis factor (TNF-α) enzyme-linked immunosorbent assay (ELISA) kit, the rat interleukin 1β (IL-1β) ELISA kit and the rat interleukin 6 (IL-6) ELISA kit were purchased from Nanjing Jiancheng Institute of Biological Engineering (lot Numbers: A20200810, A20200807, A20200816). The caspase inhibitor was benzyloxycarbonyl-L-aspart-1-yl-[2,6-dichlorobenzoyl]methane (Z-Asp-CH2-DCB) (Cayman, lot numbers: 15143-10).

A TDZ5-WS centrifuge (Hunan Xiangyi Centrifuge Instrument Co., Ltd.), a BSA224S type analytical balance (Sartorius, Germany), a VICTOR X3 enzyme marker (PerkinElmer, USA), and an i-STAT clinical hematology analyzer (ABBOTT, USA) were used.

### Establishment of animal model and grouping of experimental animals

**Figure 1:**
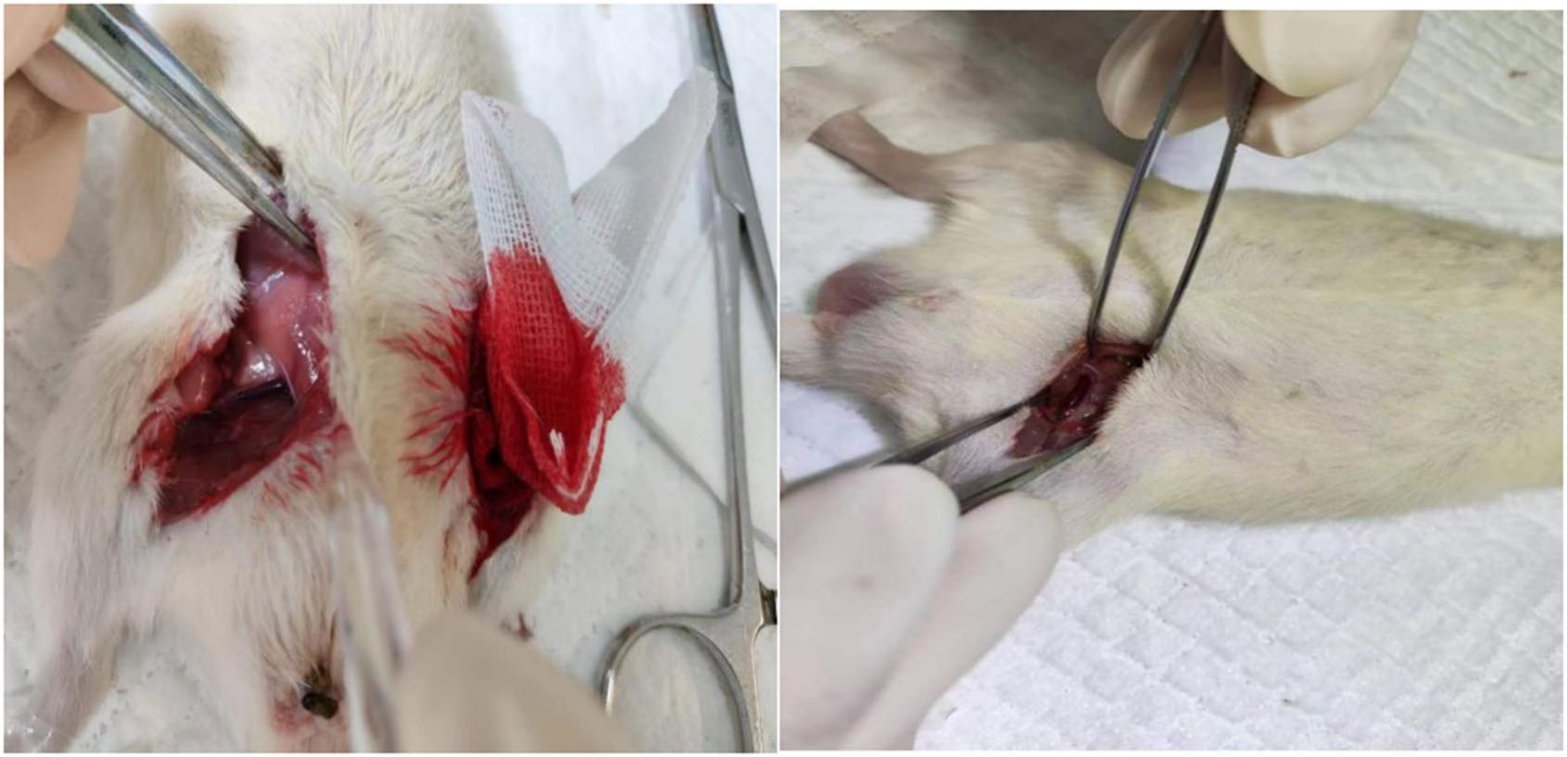
Measurement of MAP and PAP through intravascular catheterization.

Sixty healthy male Wistar rats were randomly divided into three groups, namely, the control group, ARDS group and ARDS group + Caspase inhibitor group, with 20 rats in each group. The control group was intraperitoneally injected with 2 mL/kg saline, and the ARDS model group was established by intraperitoneally injecting 4 mg/kg LPS^13,14^. Drug administration group was adminstered 20 mg/kg caspase inhibitor after intraperitoneal LPS injection. Behavioral changes in the rats were observed after modeling.Rats were i.p. anesthetized using pentobarbital injections. A 1-mm diameter polyethylene plastic microcatheter was inserted from the right external jugular vein of rats according to a previously reported method in the literature^15,16^, and the changes in PAP and systemic arterial blood pressure at 6 and 12 h were observed (Fig. 1). Six hours after modeling, blood was collected from the rat’s abdominal aorta with a blood gas needle to collect arterial blood, and arterial blood gas was immediately analyzed by an i-STAT clinical blood gas analyzer to compare the PaO_2_, PaCO_2_ and PaO_2_/FiO_2_ of the two groups. Thirty minutes later, the rats in each group were anesthetized with 0.4% pentobarbital sodium solution (40 mg/kg, i.p.) and painlessly sacrificed by bloodletting from the right femoral artery. Finally, the thoracic cavity was opened, the intact lung was separated and removed, the right lung was ligated, and the tissue of the lower lobe of the right lung was removed to determine the W/D ratio. One hundred milligrams of lung tissue was taken and homogenized by adding saline, and the levels of TNF-α, IL-1β and IL-6 in the lung tissue homogenate were measured by ELISA kits.

### Morphological observation of pulmonary vascular endothelial cells

Ten rats in each group were randomly selected and the right lung tissue was harvested after sacrifice. The remaining blood was washed off with saline and then the lung tissue was perfused with “1% formalin + 0.5% agarose” at 20 cm water column (cmH_2_O) pressure. The tissue was then fixed with 10% neutral formaldehyde solution for 1 day, paraffin-embedded and serially sectioned. Four lung tissue sections were selected from each rat and subjected to hematoxylin–eosin (HE), Elastin-van Gieson and α-actin immunohistochemical staining to observe the following: ①MT%: HE staining and Elastin-van Gieson staining were observed under a 400 × light microscope. Within the Van Gieson stained sections, 4 small pulmonary arteries (15–100 μm diameter) were randomly selected from each section, and the mean diameters of the internal and external elastic plates were determined using the SimplePCI image analysis software system to calculate the intima-media thickness (MT) and MT% of small pulmonary arteries = (2 × intima-media thickness)/external diameter × 100%; ② Degree of myosinization of unmyelinated vessels: α-actin immunohistochemically stained sections were observed under 400 × light microscope, 10 high magnification fields were randomly observed in each section, small pulmonary arteries with diameters of 15–50 μm (30) were counted, and positive α-actin staining of the vessel wall was considered as myosinization, and the degree of myosinization of unmyelinated vessels = (complete myosinization + number of partially myosinized vessels)/total number of observed vessels × 100%.

### Statistical methods

SPSS 19.0 software was used for statistical analysis of the data. All experimental data are expressed as $\overline{X}$ ± SD. A t test was applied for comparison of the mean between two groups, and differences were considered statistically significant at *P* < 0.05.

## Results

### Changes in behavior after modeling

The control group rats had shiny hair and no signs of cyanosis were observed. There was no bloody foam-like fluid in the mouth or nasal cavity, and the rats were eating and drinking normally. After i.p. injection of LPS, the rats in the model group showed an accelerated respiratory rate, wheezing and cyanosis. Moreover, their hair was disheveled, they were not eating and drinking, and some of them had bloody foam-like liquid coming out of their mouths and nasal cavities. Over time, the state of model rats changed from irritable to depressed and drowsy, and the response to external stimuli was weakened.

### Changes in PAP and mean arterial pressure (MAP)

PAP and MAP are of great value for the diagnosis and treatment of ARDS and are important indexes for clinical monitoring. The PAP and MAP data at each time point after i.p. injection of LPS are shown in Table 1.

**Table 1:** Pulmonary artery pressure (PAP) and mean arterial pressure (MAP) in three groups of rats (n = 20, $\overline{{\text{x}}}$ ± SD).

Group	PAP (mmHg)		MAP (mmHg)	
	6 h	12 h	6 h	12 h
Control group	12.66 ± 1.26	12.74 ± 1.43	105.24 ± 5.79	105.68 ± 5.81
Model group	22.89 ± 3.63**	21.08 ± 3.62**	122.68 ± 5.82*	117.53 ± 6.26
Drug administration group	16.17 ± 1.86^##^	15.00 ± 1.62^##^	108.39 ± 5.76^#^	113.63 ± 6.13

Comparison between Model Group and Control Group, **P < 0.01, *P < 0.05; Comparison between Model Group and Drug administration group, ^##^*P* < 0.01, ^#^*P* < 0.05.

As seen from the table, compared with that of the control group rats, the PAP of the model group rats was significantly higher at each time point (*P* < 0.01) and slightly decreased at 12 h. Moreover, compared with that of the control group, the MAP of the model group was significantly higher (*P* < 0.05) at 6 h, but was not significantly different at 12 h.

Compared with the model group, drug administration group exhibited a significantly lower PAP at all time points after i.p. injection of caspase inhibitor (*P* < 0.01). The MAP was significantly lower at 6 h (*P* < 0.05), and there was no significant difference in MAP at 12 h in drug administration group compared with the model group.

### Changes in arterial blood gas

PaO_2_, PaCO_2_ and PaO_2_/FiO_2_ are important indicators to evaluate the severity of ARDS. The arterial blood gas analysis of the model and control groups is shown in Table 2. The results showed that PaO_2_, PaCO_2_ and PaO_2_/FiO_2_ were significantly lower in the model group than in the control group (*P* < 0.01). PaO_2_, PaCO_2_ and PaO_2_/FiO_2_ were significantly higher in drug administration group than in the model group (*P* < 0.05 or 0.01).

**Table 2:** Arterial blood gas indexes and lung tissue wet/dry weight (W/D) ratio in three groups of rats (n = 20, $\overline{{\text{x}}}$ ± SD).

Group	PaO_2_ (mmHg)	PaCO_2_ (mmHg)	PaO_2_/FiO_2_	W/D (g/g)
Control group	85.81 ± 5.74	41.51 ± 4.60	428.24 ± 7.67	2.78 ± 0.08
Model group	41.84 ± 5.35**	22.17 ± 5.94**	289.76 ± 7.88**	5.12 ± 0.17**
Drug administration group	71.69 ± 5.68^##^	31.67 ± 5.26^#^	399.13 ± 7.79^##^	3.87 ± 0.16^#^

Comparison between Model Group and Control Group, **P < 0.01; Comparison between Model Group and Drug administration group, ^##^ *P* < 0.01, ^#^ *P* < 0.05.

### Changes in the lung tissue W/D ratio

The W/D ratio of lung tissue is an important indicator of lung permeability, and the W/D ratios of the control and model groups are shown in Table 2. As seen from the table, the W/D ratio of rats in the LPS model group was significantly higher than that of the control group (*P* < *0*.01). The W/D ratio of rats in the drug administration group was significantly lower than that of the model group (*P* < 0.05).

### Changes in inflammatory factor levels in lung tissue

After LPS stimulation, a large amount of pro-inflammatory mediators (TNF-α, IL-1β, IL-6, etc.) and chemokines (CXCL-1, CXCL-2, etc.) are released in the lung to induce an acute inflammatory response in the organism, and the changes in inflammatory factor levels in the lung tissue homogenates of the two groups of rats are shown in Table 3. As seen from the table, the levels of TNF-α, IL-1β and IL-6 in the LPS model group were significantly higher than those in the control group (*P* < *0*.01). Compared with those in the model group, the levels of TNF-α, IL-1β and IL-6 in drug administration group were significantly lower (*P* < 0.01).

**Table 3:** Inflammatory factor levels in three groups of rats (n = 20, $\overline{{\text{x}}}$ ± SD).

Group	TNF-α (pg/mL)	IL-1β (pg/mL)	IL-6 (pg/mL)
Control group	49.88 ± 1.82	108.81 ± 7.37	41.15 ± 3.51
Model group	346.79 ± 5.76**	796.33 ± 32.56**	349.45 ± 17.35**
Drug administration group	216.98 ± 3.98^##^	489.61 ± 24.36^##^	209.31 ± 16.98^##^

Comparison between Model Group and Control Group, **P < 0.01; Comparison between Model Group and Drug administration group, ^##^*P* < 0.01.

### Morphological observation of pulmonary vascular endothelial cells

**Figure 2:**
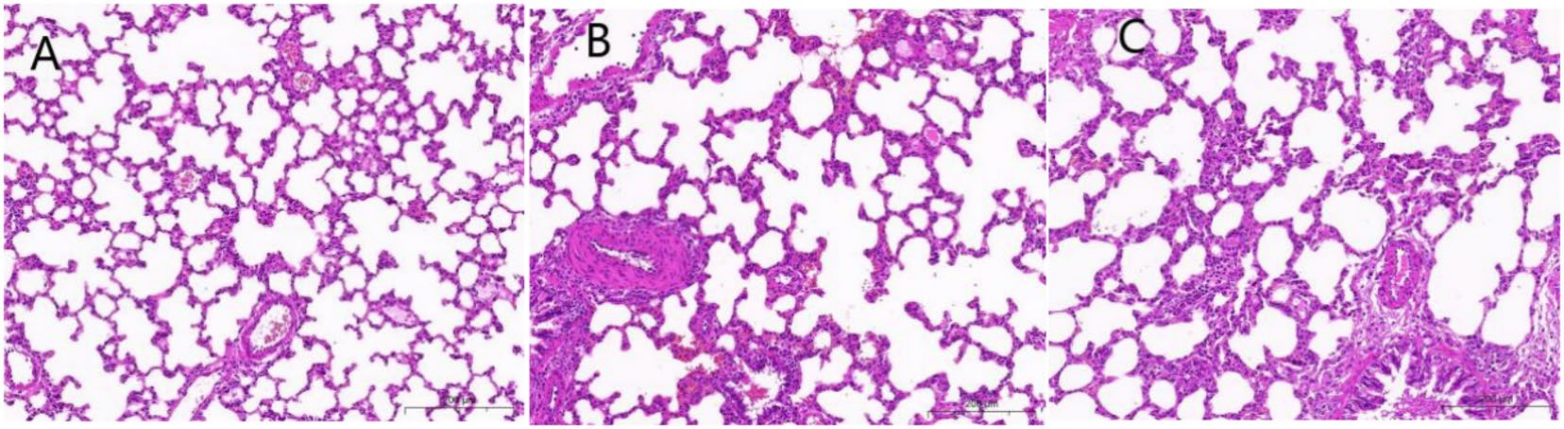
Comparison of pathological characteristics among groups. (**A**) Control group, (**B**) Model group, (**C**) Drug administration group.

After HE staining, it was observed that compared with the control group, the model group had significantly higher (*P* < 0.05 or 0.01) MT and MT% of small pulmonary arteries and significantly higher (*P* < 0.01) muscle vascularization of unmyelinated vessels. Compared with that of the model group, the MT of small pulmonary arteries in drug administration group was significantly lower (*P* < 0.01), and the degree of myosinization in unmyelinated vessels was significantly lower (*P* < *0*.05). The results are shown in Table 4 and Fig. 2.

**Table 4:** Intima-media thickness (MT) and muscle vascularization of the small pulmonary arteries in three groups of rats.

Group	MT (μm)	MT%	Degree of muscle vascularization (%)
Control group	10.87 ± 0.84	21.11 ± 7.37	5.01 ± 0.35
Model group	43.51 ± 2.45**	26.33 ± 4.56*	9.27 ± 0.64**
Drug administration group	28.65 ± 1.66^##^	22.69 ± 6.98	7.19 ± 0.54^#^

Comparison between Model Group and Control Group, **P < 0.01, *P < 0.05; Comparison between Model Group and Drug administration group, ^##^*P* < 0.01, ^#^*P* < 0.05.

## Discussion

ARDS is caused by a variety of factors, including sepsis, pneumonia, trauma, acute pancreatitis, and gastric content aspiration^17,18^. ARDS is characterized by diffuse alveolar injury, pulmonary edema, neutrophil-derived inflammation, and surface-active substance dysfunction^19^. Its clinical manifestations include reduced pulmonary compliance, severe hypoxemia and bilateral pulmonary infiltrates. Apoptosis, also known as programmed cell death (PCD), is mediated by the caspase family.The activated caspase family causes apoptosis by triggering a cascade reactions of enzymes,which is involved in the pathogenesis of ARDS^20^, while blocking the caspase cascade can alleviate ARDS^21–23^. ARC is a recently discovered unique endogenous inhibitor of apoptosis with efficient and multifunctional effects. At present, research on the function of ARC mainly focuses on cancer and heart diseases, and studies show that ARC plays an anti-apoptotic role by regulating the interaction between ARC and caspase or its receptor proteins, including the specific inhibition of caspase-2 and caspase-8 activity^24,25^, which can antagonize apoptosis caused by hypoxia and so on^26^.

The pathophysiological basis of PH in ARDS is pulmonary capillary occlusion, vascular remodeling, and increased progressive pulmonary vascular resistance (PVR), which can lead to pulmonary circulatory dysfunction, acute pulmonary heart disease (ACP) and right heart failure and is an independent risk factor for morbidity and mortality in patients with ARDS^27^. There is no unified standard for the diagnosis of PH, and its diagnosis is mainly based on cardiac ultrasonography combined with clinical symptoms^28,29^. Modern studies have shown that hypoxia-induced apoptosis of pulmonary vascular endothelial cells (PVECs) is the initial step in the development of PH^30^. Hypoxic conditions directly trigger pulmonary vasoconstriction and affect right heart function, while they also play an important role in pulmonary vascular remodeling^31^. In this study, the ARDS model was established by i.p. injection of LPS and then the model rats were treated with ARC. The PAP and MAP were measured at 6 and 12 h after modeling. After drug administration, the PAP was significantly lower in the drug administration group than in the model group at all time points, and the MAP was significantly lower at 6 h. From the above results, it can be concluded that this caspase inhibitor can effectively regulate PH hemodynamics in ARDS model rats. Wei et al.^32^ found that SU5416 combined with hypoxia can aggravate pulmonary hypertension and pulmonary vascular remodeling in rats. Therefore, preventing PVECs apoptosis by inhibiting caspase activation in the early stage of ARDS may be an important strategy to alleviate PH and inhibit pulmonary vascular remodeling. It is worth to further study.

The damage of alveolar-capillary barrier plays an important role in the development of ARDS,because it leads to flooding of the alveolar spaces with protein-rich exudates. Apoptosis of alveolar epithelial cells is an important mechanism leading to this injury^33^. Blood gas analysis showed that PaO_2_, PaCO_2_ and PaO_2_/FiO_2_ were significantly lower in the model group than in the control group and that PaO_2_, PaCO_2_ and PaO_2_/FiO_2_ were significantly higher after drug administration.It is suggested that ARC intervention may improve the alveolar-capillary barrier function and help to avoid further deterioration of pulmonary oxygenation.

The inflammatory response plays an important role in ARDS, and the increased release of proinflammatory factors and the relatively insufficient amount of anti-inflammatory factors in the early stage of ARDS are the main mechanisms leading to an uncontrolled inflammatory response in ARDS^34–36^. A previous study showed that the lung tissue of LPS-induced ARDS model rats exhibited increased infiltration of polymorphonuclear leukocytes (PMNs), which produce and release many inflammatory factors, including IL-1α, IL-1β, IL-4, IL-6, IL-10, and TNF-α^37^. Among them, TNF-α, as the initiating factor of ARDS, represents the severity of the inflammatory response in the early stage of ARDS^38^. Hypoxia and inflammation in patients with ARDS experience hypertrophy of smooth muscle in the distal lung vessels, myelin dysregulation and thickened intima, which eventually leads to pulmonary vascular morphology remodeling, causing an increase in PVR and the formation of PH^39^. Agrawal et al. observed through animal experiments that a hypoxic environment can directly upregulate IL-6, which plays an important role in the process of pulmonary vascular remodeling and PH formation. The proinflammatory cytokines IL-1β and IL-6 can play a major role in the occurance and progression of ARDS by stimulating neutrophil chemotaxis^40^. The present study showed that the levels of inflammatory mediators (TNF-α, IL-1β and IL-6) were significantly elevated in the LPS model group and significantly decreased after ARC treatment. From these results, it can be concluded that ARC, a caspase inhibitor, can effectively reduce the levels of inflammatory factors in ARDS model rats.

In this study, we only observed the effect of caspase inhibitor on ARDS model rats, but we did not study the mechanism of action in depth, and we need to explore the mechanism of action in subsequent studies. In addition, we have only done in vivo studies of cells, and have not gone deeper for in vitro cellular studies to explore further roles and possible mechanisms.

In conclusion, this study revealed the effects of a caspase inhibitor on PH, hemodynamics, and inflammatory factors in a rat model of ARDS, providing a theoretical basis for the treatment of PH in clinical ARDS patients.

## Data Availability

All data generated or analyzed during this study are included in this article. Further enquiries can be directed to the corresponding author.
